# 1-[3,5-Bis(trifluoro­meth­yl)phen­yl]-3-(2-pyrid­yl)thio­urea

**DOI:** 10.1107/S1600536808009768

**Published:** 2008-04-16

**Authors:** Huadong Yue, Yifeng Wang, Aibao Xia, Shuping Luo, Danqian Xu

**Affiliations:** a State Key Laboratory Breeding Base of Green Chemistry-Synthesis Technology, Zhejiang University of Technology, Hangzhou 310014, People’s Republic of China

## Abstract

The title compound, C_14_H_9_F_6_N_3_S, exhibits a nearly planar conformation in the solid state, with a dihedral angle between the planes of the benzene and pyridine rings of 14.86 (3)°. The pyridine N atom allows for the formation of a six-membered N—H⋯N_py_ hydrogen-bonded ring, thus forcing the two amide H atoms of the thio­urea group to point in opposite directions. The second N—H group forms an inter­molecular N—H⋯S hydrogen bond with the S atom of an adjacent mol­ecule. The F atoms of the two trifluoro­methyl groups display rotational disorder around the C—CF_3_ axis, with an occupancy ratio of 0.54 (1):0.46 (1).

## Related literature

For related literature, see: Akiyama *et al.* (2006 ▶); Struga *et al.* (2007 ▶).
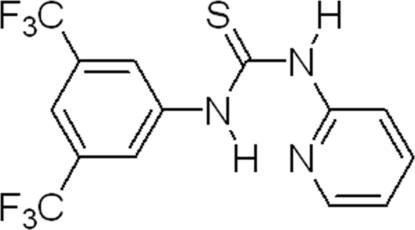


## Experimental

### 

#### Crystal data

C_14_H_9_F_6_N_3_S
*M* *_r_* = 365.30
Orthorhombic, 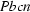

*a* = 15.0907 (17) Å
*b* = 7.7491 (9) Å
*c* = 26.709 (3) Å
*V* = 3123.3 (6) Å^3^
*Z* = 8
Mo *K*α radiation
μ = 0.27 mm^−1^
*T* = 293 (2) K
0.41 × 0.31 × 0.17 mm

#### Data collection

Bruker SMART CCD area-detector diffractometer
Absorption correction: multi-scan (*SADABS*; Sheldrick, 1996 ▶) *T*_min_ = 0.873, *T*_max_ = 0.962
17235 measured reflections
3406 independent reflections
2585 reflections with *I* > 2σ(*I*)
*R*_int_ = 0.102

#### Refinement

*R*[*F* ^2^ > 2σ(*F* ^2^)] = 0.058
*wR*(*F* ^2^) = 0.151
*S* = 1.06
3406 reflections
280 parameters
13 restraints
H atoms treated by a mixture of independent and constrained refinement
Δρ_max_ = 0.40 e Å^−3^
Δρ_min_ = −0.30 e Å^−3^

### 

Data collection: *SMART* (Bruker, 2001 ▶); cell refinement: *SAINT-Plus* (Bruker, 2000 ▶); data reduction: *SAINT-Plus* and *SHELXTL* (Sheldrick, 2008 ▶); program(s) used to solve structure: *SHELXS97* (Sheldrick, 2008 ▶); program(s) used to refine structure: *SHELXL97* (Sheldrick, 2008 ▶); molecular graphics: *SHELXTL*; software used to prepare material for publication: *SHELXTL* and *PLATON* (Spek, 2003 ▶).

## Supplementary Material

Crystal structure: contains datablocks global, I. DOI: 10.1107/S1600536808009768/zl2103sup1.cif

Structure factors: contains datablocks I. DOI: 10.1107/S1600536808009768/zl2103Isup2.hkl

Additional supplementary materials:  crystallographic information; 3D view; checkCIF report

## Figures and Tables

**Table 1:** Hydrogen-bond geometry (Å, °)

*D*—H⋯*A*	*D*—H	H⋯*A*	*D*⋯*A*	*D*—H⋯*A*
N2—H2⋯N3	0.83 (2)	1.92 (2)	2.641 (3)	144 (2)
N1—H1⋯S1^i^	0.798 (17)	2.617 (18)	3.3931 (19)	165 (3)

Symmetry code: (i) 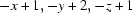.

**Fig. 1.**
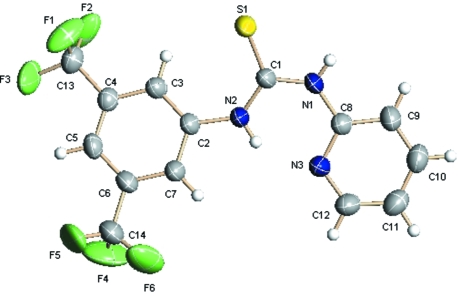
View of the structure of the title compound showing the atom-labeling scheme. Displacement ellipsoids are drawn at the 50% probability level. For the sake of clarity, only the major orientation of the CF3 group is shown.

**Fig. 2.**
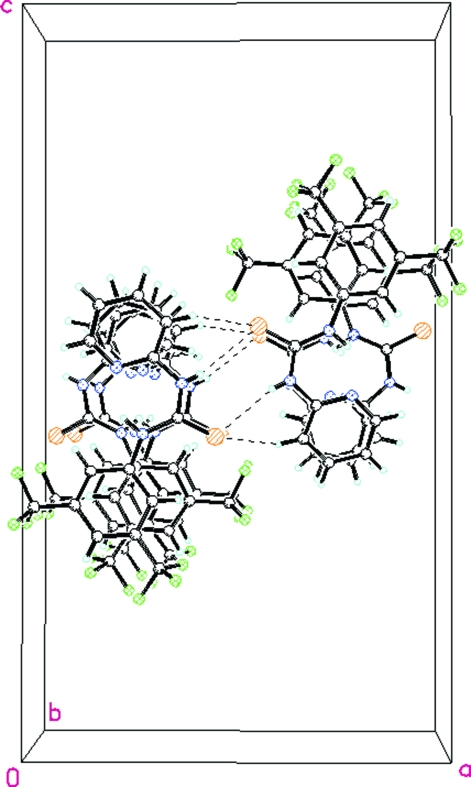
The molecular packing of the title compound showing the intermolecular N—H···S and C—H···S hydrogen-bonding interactions (dashed lines).

## supplementary crystallographic information

### Comment

Thiourea compounds have been extensively studied over the last few years due to their pharmacological and biological activities (Struga *et al.*, 2007). Recently, excellent results have been also achieved with the use of the bifunctional thiourea catalysts, which effectively activate carbonyl groups and imines through double hydrogen-bonding interactions in asymmetric synthesis (Akiyama *et al.*, 2006). Herein, we synthesized the title compound (I) and determined its crystal structure (Fig.1). The benzene and pyridine rings in the structural unit of (I) are almost perfectly coplanar with a dihedral angle between their planes of only 14.86 (3)°. Like other 2-pyridyl thioureas, the title compound exhibits both intramolecular and intermolecular hydrogen bonding interactions. The pyridine nitrogen N3 allows for the formation of a six membered N—H···N_pyr_ hydrogen bonded ring which forces the two amide hydrogen atoms of the thiourea group to point in opposite directions. At the same time, the N1—H1 and C9—H9 moieties of the pyridine ring form intermolecular N—H···S and C—H···S hydrogen bonds with the S atom of an adjacent molecule (Fig. 2). This weak hydrogen-bonding network leads to the formation of infinite chains of molecules thus stabilizing the crystal packing.

### Experimental

The title compound was synthesized by treating 3,5-bis-trifluoromethyl-phenyl isothiocyanate (2.71 g, 10 mmol) with 2-amino pyridine (0.94 g, 10 mmol) in MeCN (30 ml) under stirring at room temperature for 24 h. Suitable crystals of the title compound were obtained by slow evaporation of an actonitrile solution at room temperature (3.28 g, 10 mmol). Yield 90%, m.p. 431–433 K, ^1^H NMR (500 MHz, CDCl_3_): 6.91 (d, J = 8 Hz, 1H), 7.08–7.11 (m, 1H), 7.73–7.77 (m, 2H), 8.27 (s, 1H), 8.29–8.31 (m, 2H), 8.95 (s, 1H), 14.17 (s, 1H).

### Refinement

Large solvent accessible voids are found in the crystal. The volumes (31 Å^3^ per cavity, four equivalent cavities per unit cell) of these voids are not quite large enough to host acetonitrile molecules, the solvent of crystallization, and no significant residual electron density is seen in difference Fourier syntheses maps. The largest residual electron density peak and the deepest negative density in these voids are 0.40 (0.97 Å from S1) and -0.30 (1.30 Å from C4), respectively. Also, an attempted correction for the electron density within the voids using the Squeeze algorithm implemented in the program PLATON (Spek, 2003) does not significantly improve the quality of the dataset or refinement. The fluorine atoms of the two CF_3_ groups exhibit conformational disorder around the C4—C13 and C6—C14 bonds with an occupancy ratio of 0.54 (1) to 0.46 (1). They are refined with restraints for the C—F bond lengths and the F···F interatomic distances to maintain nearly tetrahedral geometry. All C—F bond lengths are restrained to 1.35 (5) Å and the displacement parameters of the disordered F atoms are restrained to an approximate isotropic behaviour. All carbon-bond H atoms are placed in calculated positions with C—H = 0.93 Å (aromatic) and refined using a riding model, with *U*_iso_(H) = 1.2_eq_(C). N-bound H atoms are located in a difference map and refined with an N—H distance restraint of 0.83 (2) Å.

### Crystal data

**Table:**

C_14_H_9_F_6_N_3_S	*D*_x_ = 1.554 Mg m^−^^3^
*M**_r_* = 365.30	Mo *K*α radiation λ = 0.71073 Å
Orthorhombic, *P**b**c**n*	Cell parameters from 4062 reflections
*a* = 15.0907 (17) Å	θ = 5.4–45.0º
*b* = 7.7491 (9) Å	µ = 0.27 mm^−^^1^
*c* = 26.709 (3) Å	*T* = 293 (2) K
*V* = 3123.3 (6) Å^3^	Prismatic, colourless
*Z* = 8	0.41 × 0.31 × 0.18 mm
*F*_000_ = 1472	

### Data collection

**Table:**

Bruker SMART CCD area-detector diffractometer	3406 independent reflections
Radiation source: fine-focus sealed tube	2585 reflections with *I* > 2σ(*I*)
Monochromator: graphite	*R*_int_ = 0.102
*T* = 293(2) K	θ_max_ = 27.0º
φ and ω scans	θ_min_ = 1.5º
Absorption correction: multi-scan(SADABS; Sheldrick, 1996)	*h* = −19→19
*T*_min_ = 0.873, *T*_max_ = 0.962	*k* = −9→9
17235 measured reflections	*l* = −26→34

### Refinement

**Table:**

Refinement on *F*^2^	Secondary atom site location: difference Fourier map
Least-squares matrix: full	Hydrogen site location: inferred from neighbouring sites
*R*[*F*^2^ > 2σ(*F*^2^)] = 0.058	H atoms treated by a mixture of independent and constrained refinement
*wR*(*F*^2^) = 0.151	*w* = 1/[σ^2^(*F*_o_^2^) + (0.075*P*)^2^ + 0.3374*P*] where *P* = (*F*_o_^2^ + 2*F*_c_^2^)/3
*S* = 1.06	(Δ/σ)_max_ = 0.040
3406 reflections	Δρ_max_ = 0.40 e Å^−^^3^
280 parameters	Δρ_min_ = −0.30 e Å^−^^3^
13 restraints	Extinction correction: none
Primary atom site location: structure-invariant direct methods	

### Special details

**Table:**

Geometry. All e.s.d.'s (except the e.s.d. in the dihedral angle between two l.s. planes) are estimated using the full covariance matrix. The cell e.s.d.'s are taken into account individually in the estimation of e.s.d.'s in distances, angles and torsion angles; correlations between e.s.d.'s in cell parameters are only used when they are defined by crystal symmetry. An approximate (isotropic) treatment of cell e.s.d.'s is used for estimating e.s.d.'s involving l.s. planes.
Refinement. Refinement of *F*^2^ against ALL reflections. The weighted *R*-factor *wR* and goodness of fit *S* are based on *F*^2^, conventional *R*-factors *R* are based on *F*, with *F* set to zero for negative *F*^2^. The threshold expression of *F*^2^ > σ(*F*^2^) is used only for calculating *R*-factors(gt) *etc*. and is not relevant to the choice of reflections for refinement. *R*-factors based on *F*^2^ are statistically about twice as large as those based on *F*, and *R*-factors based on ALL data will be even larger.

### Fractional atomic coordinates and isotropic or equivalent isotropic displacement parameters (Å^2^)

**Table:**

	*x*	*y*	*z*	*U*_iso_*/*U*_eq_	Occ. (<1)
S1	0.44883 (4)	1.05153 (11)	0.42753 (2)	0.0699 (3)	
N1	0.37456 (12)	0.8647 (3)	0.49769 (7)	0.0499 (5)	
N2	0.28358 (12)	0.9220 (3)	0.43161 (6)	0.0468 (5)	
N3	0.22735 (11)	0.7940 (2)	0.51757 (6)	0.0480 (5)	
C1	0.36381 (13)	0.9414 (3)	0.45239 (8)	0.0456 (5)	
C2	0.25117 (14)	0.9797 (3)	0.38500 (7)	0.0406 (5)	
C3	0.30288 (14)	1.0050 (3)	0.34296 (7)	0.0435 (5)	
H3	0.3640	0.9898	0.3445	0.052*	
C4	0.26236 (15)	1.0534 (3)	0.29841 (7)	0.0426 (5)	
C5	0.17215 (15)	1.0763 (3)	0.29537 (8)	0.0477 (5)	
H5	0.1459	1.1092	0.2653	0.057*	
C6	0.12109 (14)	1.0495 (3)	0.33762 (8)	0.0472 (5)	
C7	0.15999 (14)	1.0010 (3)	0.38225 (8)	0.0451 (5)	
H7	0.1251	0.9825	0.4104	0.054*	
C8	0.31247 (13)	0.7912 (3)	0.53057 (7)	0.0431 (5)	
C9	0.34218 (16)	0.7222 (3)	0.57550 (8)	0.0538 (6)	
H9	0.4023	0.7189	0.5830	0.065*	
C10	0.28091 (17)	0.6595 (3)	0.60830 (9)	0.0575 (6)	
H10	0.2989	0.6127	0.6387	0.069*	
C11	0.19245 (17)	0.6658 (3)	0.59626 (9)	0.0597 (6)	
H11	0.1496	0.6260	0.6184	0.072*	
C12	0.16964 (15)	0.7320 (3)	0.55103 (9)	0.0565 (6)	
H12	0.1098	0.7343	0.5427	0.068*	
C13	0.31954 (17)	1.0781 (3)	0.25314 (8)	0.0550 (6)	
C14	0.02309 (17)	1.0700 (4)	0.33485 (10)	0.0693 (8)	
F1	0.3600 (6)	1.2197 (7)	0.2520 (3)	0.092 (3)	0.540 (15)
F2	0.3770 (6)	0.9559 (14)	0.2476 (4)	0.106 (4)	0.540 (15)
F3	0.2703 (4)	1.0759 (17)	0.21099 (16)	0.105 (3)	0.540 (15)
F4	−0.0158 (11)	0.927 (2)	0.3219 (7)	0.114 (5)	0.540 (15)
F5	−0.0038 (9)	1.1890 (19)	0.3061 (5)	0.111 (6)	0.540 (15)
F6	−0.0122 (7)	1.1014 (17)	0.3796 (3)	0.118 (4)	0.540 (15)
F1'	0.3995 (6)	1.140 (2)	0.2647 (2)	0.112 (5)	0.460 (15)
F2'	0.3344 (11)	0.9413 (13)	0.2292 (4)	0.116 (5)	0.460 (15)
F3'	0.2887 (7)	1.1881 (17)	0.2217 (4)	0.117 (5)	0.460 (15)
F4'	−0.0137 (13)	0.957 (3)	0.3042 (8)	0.115 (7)	0.460 (15)
F5'	0.0011 (10)	1.2244 (16)	0.3174 (6)	0.111 (4)	0.460 (15)
F6'	−0.0171 (6)	1.0587 (17)	0.3773 (3)	0.097 (4)	0.460 (15)
H1	0.4219 (13)	0.877 (3)	0.5105 (9)	0.065 (8)*	
H2	0.2456 (16)	0.882 (3)	0.4511 (9)	0.047 (6)*	

### Atomic displacement parameters (Å^2^)

**Table:**

	*U*^11^	*U*^22^	*U*^33^	*U*^12^	*U*^13^	*U*^23^
S1	0.0397 (3)	0.1245 (7)	0.0456 (4)	−0.0239 (3)	−0.0094 (2)	0.0228 (3)
N1	0.0327 (9)	0.0801 (14)	0.0370 (9)	−0.0038 (9)	−0.0087 (7)	0.0105 (9)
N2	0.0334 (9)	0.0741 (13)	0.0328 (9)	−0.0062 (8)	−0.0037 (7)	0.0073 (8)
N3	0.0371 (9)	0.0676 (12)	0.0394 (9)	−0.0050 (8)	−0.0015 (7)	0.0030 (8)
C1	0.0331 (10)	0.0688 (15)	0.0350 (11)	−0.0001 (9)	−0.0020 (8)	0.0014 (9)
C2	0.0369 (10)	0.0511 (12)	0.0337 (10)	−0.0034 (9)	−0.0056 (8)	−0.0019 (8)
C3	0.0377 (10)	0.0552 (12)	0.0376 (11)	0.0001 (9)	−0.0011 (8)	−0.0029 (9)
C4	0.0482 (12)	0.0452 (12)	0.0345 (10)	−0.0035 (9)	−0.0018 (9)	−0.0024 (8)
C5	0.0523 (13)	0.0542 (13)	0.0368 (11)	−0.0019 (10)	−0.0104 (9)	0.0051 (9)
C6	0.0369 (11)	0.0579 (14)	0.0468 (12)	−0.0021 (9)	−0.0095 (9)	0.0039 (10)
C7	0.0372 (11)	0.0609 (13)	0.0371 (11)	−0.0041 (9)	−0.0025 (8)	0.0031 (9)
C8	0.0405 (11)	0.0522 (12)	0.0367 (10)	−0.0028 (9)	−0.0020 (8)	−0.0010 (9)
C9	0.0520 (13)	0.0655 (15)	0.0440 (12)	−0.0024 (11)	−0.0084 (10)	0.0098 (10)
C10	0.0692 (16)	0.0621 (15)	0.0410 (12)	−0.0048 (12)	−0.0018 (11)	0.0076 (10)
C11	0.0642 (15)	0.0647 (16)	0.0502 (14)	−0.0094 (12)	0.0109 (11)	0.0040 (11)
C12	0.0433 (12)	0.0751 (17)	0.0511 (13)	−0.0084 (11)	0.0034 (10)	0.0018 (11)
C13	0.0656 (16)	0.0613 (16)	0.0380 (12)	−0.0007 (13)	0.0038 (10)	0.0004 (11)
C14	0.0443 (14)	0.103 (2)	0.0601 (17)	−0.0011 (15)	−0.0095 (12)	0.0122 (16)
F1	0.127 (6)	0.066 (3)	0.082 (5)	−0.042 (3)	0.050 (4)	−0.018 (2)
F2	0.124 (6)	0.103 (7)	0.092 (5)	0.055 (6)	0.063 (4)	0.033 (5)
F3	0.103 (3)	0.140 (9)	0.0328 (17)	−0.036 (5)	−0.0070 (17)	0.001 (3)
F4	0.051 (5)	0.135 (7)	0.157 (13)	−0.041 (4)	−0.005 (6)	−0.035 (8)
F5	0.058 (4)	0.147 (15)	0.089 (5)	0.026 (7)	−0.018 (3)	0.067 (7)
F6	0.062 (5)	0.144 (8)	0.097 (6)	0.044 (4)	0.004 (3)	−0.038 (5)
F1'	0.081 (5)	0.151 (15)	0.065 (3)	−0.077 (6)	0.010 (3)	0.009 (6)
F2'	0.148 (12)	0.070 (5)	0.088 (7)	−0.011 (7)	0.077 (7)	−0.023 (5)
F3'	0.113 (9)	0.121 (8)	0.077 (6)	0.054 (7)	0.040 (6)	0.066 (6)
F4'	0.062 (7)	0.147 (14)	0.126 (11)	0.003 (7)	−0.056 (7)	−0.076 (10)
F5'	0.056 (5)	0.098 (5)	0.140 (11)	0.031 (3)	0.005 (5)	0.042 (5)
F6'	0.035 (3)	0.138 (7)	0.088 (6)	−0.008 (4)	−0.002 (3)	0.066 (6)

### Geometric parameters (Å, °)

**Table:**

S1—C1	1.678 (2)	C8—C9	1.388 (3)
N1—C1	1.358 (3)	C9—C10	1.363 (3)
N1—C8	1.405 (3)	C9—H9	0.9300
N1—H1	0.798 (17)	C10—C11	1.374 (3)
N2—C1	1.340 (3)	C10—H10	0.9300
N2—C2	1.410 (3)	C11—C12	1.357 (3)
N2—H2	0.83 (2)	C11—H11	0.9300
N3—C8	1.331 (3)	C12—H12	0.9300
N3—C12	1.337 (3)	C13—F1	1.256 (5)
C2—C3	1.381 (3)	C13—F2'	1.259 (8)
C2—C7	1.388 (3)	C13—F3'	1.285 (5)
C3—C4	1.389 (3)	C13—F2	1.292 (7)
C3—H3	0.9300	C13—F1'	1.335 (6)
C4—C5	1.375 (3)	C13—F3	1.349 (5)
C4—C13	1.498 (3)	C14—F5	1.268 (9)
C5—C6	1.382 (3)	C14—F6'	1.288 (8)
C5—H5	0.9300	C14—F4	1.301 (13)
C6—C7	1.381 (3)	C14—F4'	1.319 (13)
C6—C14	1.489 (3)	C14—F5'	1.326 (10)
C7—H7	0.9300	C14—F6	1.331 (8)
			
C1—N1—C8	130.91 (18)	N3—C12—C11	124.4 (2)
C1—N1—H1	116 (2)	N3—C12—H12	117.8
C8—N1—H1	112.3 (19)	C11—C12—H12	117.8
C1—N2—C2	130.04 (19)	F1—C13—F2'	129.6 (6)
C1—N2—H2	113.9 (16)	F1—C13—F3'	65.2 (5)
C2—N2—H2	115.5 (16)	F2'—C13—F3'	106.9 (6)
C8—N3—C12	116.63 (19)	F1—C13—F2	108.1 (6)
N2—C1—N1	115.33 (19)	F3'—C13—F2	130.9 (4)
N2—C1—S1	125.73 (17)	F2'—C13—F1'	105.0 (6)
N1—C1—S1	118.94 (15)	F3'—C13—F1'	103.9 (6)
C3—C2—C7	120.02 (18)	F2—C13—F1'	71.5 (7)
C3—C2—N2	124.51 (19)	F1—C13—F3	104.9 (4)
C7—C2—N2	115.35 (18)	F2'—C13—F3	70.3 (6)
C2—C3—C4	119.1 (2)	F2—C13—F3	105.4 (5)
C2—C3—H3	120.5	F1'—C13—F3	134.0 (4)
C4—C3—H3	120.5	F1—C13—C4	114.3 (3)
C5—C4—C3	121.40 (19)	F2'—C13—C4	113.9 (5)
C5—C4—C13	120.41 (19)	F3'—C13—C4	113.8 (3)
C3—C4—C13	118.2 (2)	F2—C13—C4	112.6 (4)
C4—C5—C6	118.98 (19)	F1'—C13—C4	112.4 (3)
C4—C5—H5	120.5	F3—C13—C4	110.8 (3)
C6—C5—H5	120.5	F5—C14—F6'	115.6 (8)
C7—C6—C5	120.6 (2)	F5—C14—F4	108.3 (11)
C7—C6—C14	119.6 (2)	F6'—C14—F4	87.9 (10)
C5—C6—C14	119.8 (2)	F5—C14—F4'	88.3 (12)
C6—C7—C2	119.97 (19)	F6'—C14—F4'	107.6 (11)
C6—C7—H7	120.0	F6'—C14—F5'	104.6 (9)
C2—C7—H7	120.0	F4—C14—F5'	124.2 (11)
N3—C8—C9	122.95 (19)	F4'—C14—F5'	105.9 (12)
N3—C8—N1	118.28 (18)	F5—C14—F6	106.4 (9)
C9—C8—N1	118.76 (19)	F4—C14—F6	102.3 (10)
C10—C9—C8	118.3 (2)	F4'—C14—F6	120.7 (11)
C10—C9—H9	120.9	F5'—C14—F6	92.8 (9)
C8—C9—H9	120.9	F5—C14—C6	115.2 (7)
C9—C10—C11	119.7 (2)	F6'—C14—C6	114.6 (5)
C9—C10—H10	120.1	F4—C14—C6	111.8 (9)
C11—C10—H10	120.1	F4'—C14—C6	112.2 (10)
C12—C11—C10	117.9 (2)	F5'—C14—C6	111.2 (7)
C12—C11—H11	121.0	F6—C14—C6	111.9 (5)
C10—C11—H11	121.0		
			
C2—N2—C1—N1	−178.0 (2)	C8—N3—C12—C11	−0.8 (4)
C2—N2—C1—S1	2.6 (4)	C10—C11—C12—N3	−1.0 (4)
C8—N1—C1—N2	−11.5 (4)	C5—C4—C13—F1	101.7 (6)
C8—N1—C1—S1	167.9 (2)	C3—C4—C13—F1	−79.2 (6)
C1—N2—C2—C3	28.6 (4)	C5—C4—C13—F2'	−93.6 (10)
C1—N2—C2—C7	−155.3 (2)	C3—C4—C13—F2'	85.6 (10)
C7—C2—C3—C4	0.7 (3)	C5—C4—C13—F3'	29.4 (9)
N2—C2—C3—C4	176.6 (2)	C3—C4—C13—F3'	−151.5 (9)
C2—C3—C4—C5	−0.1 (3)	C5—C4—C13—F2	−134.4 (7)
C2—C3—C4—C13	−179.3 (2)	C3—C4—C13—F2	44.7 (7)
C3—C4—C5—C6	−0.3 (3)	C5—C4—C13—F1'	147.1 (10)
C13—C4—C5—C6	178.9 (2)	C3—C4—C13—F1'	−33.7 (10)
C4—C5—C6—C7	0.1 (3)	C5—C4—C13—F3	−16.6 (7)
C4—C5—C6—C14	−178.7 (2)	C3—C4—C13—F3	162.5 (6)
C5—C6—C7—C2	0.4 (3)	C7—C6—C14—F5	145.4 (9)
C14—C6—C7—C2	179.3 (2)	C5—C6—C14—F5	−35.8 (10)
C3—C2—C7—C6	−0.8 (3)	C7—C6—C14—F6'	7.6 (8)
N2—C2—C7—C6	−177.1 (2)	C5—C6—C14—F6'	−173.6 (7)
C12—N3—C8—C9	2.4 (3)	C7—C6—C14—F4	−90.4 (9)
C12—N3—C8—N1	−176.2 (2)	C5—C6—C14—F4	88.4 (9)
C1—N1—C8—N3	−0.6 (4)	C7—C6—C14—F4'	−115.6 (12)
C1—N1—C8—C9	−179.3 (2)	C5—C6—C14—F4'	63.3 (12)
N3—C8—C9—C10	−2.1 (4)	C7—C6—C14—F5'	126.0 (8)
N1—C8—C9—C10	176.5 (2)	C5—C6—C14—F5'	−55.2 (8)
C8—C9—C10—C11	0.1 (4)	C7—C6—C14—F6	23.7 (7)
C9—C10—C11—C12	1.4 (4)	C5—C6—C14—F6	−157.5 (7)

### Hydrogen-bond geometry (Å, °)

**Table:**

*D*—H···*A*	*D*—H	H···*A*	*D*···*A*	*D*—H···*A*
N2—H2···N3	0.83 (2)	1.92 (2)	2.641 (3)	144 (2)
N1—H1···S1^i^	0.798 (17)	2.617 (18)	3.3931 (19)	165 (3)

Symmetry codes: (i) −*x*+1, −*y*+2, −*z*+1.

## References

[bb1] Akiyama, T., Itoh, J. & Fuchibe, K. (2006). *Adv. Synth. Catal.* **348**, 999–1010.

[bb2] Bruker (2000). *SAINT-Plus* Bruker AXS Inc., Madison, Wisconsin, USA.

[bb3] Bruker (2001). *SMART* Bruker AXS Inc., Madison, Wisconsin, USA.

[bb6] Sheldrick, G. M. (1996). *SADABS* University of Göttingen, Germany.

[bb7] Sheldrick, G. M. (2008). *Acta Cryst.* A**64**, 112–122. 10.1107/S0108767307043930 18156677

[bb8] Spek, A. L. (2003). *J. Appl. Cryst.* **36**, 7–13.

[bb9] Struga, M., Kossakowski, J., Kedzierska, E., Fidecka, S. & Stefanska, J. (2007). *Chem. Pharm. Bull.* **55**, 796–799. 10.1248/cpb.55.796 17473472

